# Breaking anisotropy limitations in thin-film lithium niobate arrayed waveguide gratings

**DOI:** 10.1038/s41377-024-01551-w

**Published:** 2024-08-23

**Authors:** Cheng Wang

**Affiliations:** Department of Electrical Engineering & State Key Laboratory of Terahertz and Millimeter Waves, City University of Hong Kong, Kowloon, Hong Kong SAR China

**Keywords:** Integrated optics, Optoelectronic devices and components

## Abstract

A universal design strategy for dispersive elements in anisotropic platforms is proposed, enabling high-performance arrayed waveguide gratings in thin-film lithium niobate that are essential for future optical communications.

In recent years, photonic integration on the thin-film lithium niobate (TFLN) platform has attracted significant interests from both industry and academia^1^. This platform combines the excellent electro-optic (EO), acousto-optic (AO), and second-order nonlinear optical properties of lithium niobate material with high-refractive-index-contrast waveguide structures, leading to substantially reduced footprints and enhanced performance compared to conventional bulk lithium niobate devices. To date, most reported ultra-high-performance EO modulators^2,3^, AO modulators^4^, and optical frequency converters^5^ have made use of X-cut TFLN crystals due to the convenience in accessing the largest nonlinear tensor components (typically along the Z axis) in an in-plane fashion. This presents tremendous opportunities for realizing large-scale multifunctional photonic chips with unprecedented system performance and functionality on a single material platform^6^.

Photons traveling on an X-cut TFLN chip, however, experience significant anisotropy along different propagation directions, unlike other common photonic platforms like silicon and silicon nitride. While this is generally not an issue in simple waveguides, as long as the bends are gradual enough to ensure adiabatic transitions between different crystal orientations, it poses substantial challenges in designing dispersive elements, where the phase accumulation needs to be meticulously designed. A prominent example is an arrayed waveguide grating (AWG), a fundamental component for multiplexing and de-multiplexing different wavelength channels in a wavelength-division-multiplexing (WDM) network^7^. The ability to separate optical signals in closely spaced wavelength channels relies on precise control of the phase delay relationship in the many arms of an AWG, which could be easily disrupted in an anisotropic platform. This challenge has thus far hindered the experimental realization of high-performance AWGs on the X-cut TFLN platform.

In the recent work published in *Light: Science & Applications*^8^, Junjie Yi and colleagues presented a universal design strategy that could effectively mitigate the impact of optical anisotropy in waveguide-based dispersive components like AWGs. Leveraging the fact that both effective (or phase) and group indices of a waveguide mode follow a squared trigonometric dependence on the in-plane propagation angle (Fig. 1b in ref. ^8^), they propose to align the AWG such that its symmetrical axis is oriented along the 45° crystal axis. In this way, the phase variation at angle *θ* due to anisotropy is effectively compensated by that at angle 90° – *θ*, leading to simple and angle-independent phase and group delay across the arrayed waveguides. Based on this “magic” 45° twist angle, the authors successfully built an anisotropy-free AWG on an in-plane anisotropic platform, which would otherwise require fine-tuning the waveguide lengths in each path. For a 4 × 400 GHz AWG, the experimentally measured insertion loss and crosstalk were 2.4 dB and −24.1 dB, respectively, significantly outperforming previously demonstrated AWGs on the TFLN platform.

**Fig. 1:**
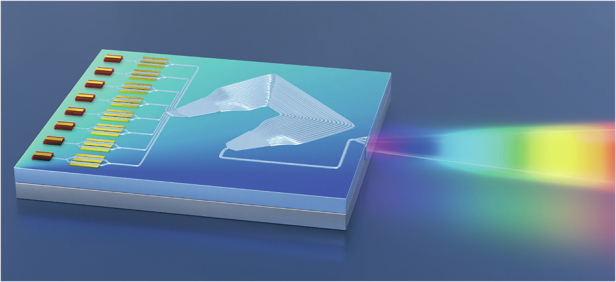
Conceptual illustration of a fully integrated WDM transmitter chip using the AWG

The successful demonstration of high-performance AWGs marks an important milestone toward many envisioned applications based on TFLN photonic integrated circuits. For example, a full WDM transmitter that integrates frequency comb sources^9^, EO modulators^2,3^, and AWGs on the same photonic chip could provide the much-needed compactness, cost-effectiveness, and ultra-high data capacity for future data center optical interconnects^10^, as the conceptual illustration in Fig. 1 shows. Further equipping the arrayed waveguides with independent EO phase shifters could enable AWGs with fast tunable wavelength channels for reconfigurable WDM and spectroscopy applications^11^.
